# How Mental Health and Suicidality Changed during the COVID-19 Pandemic: A Longitudinal Study in the General and Psychiatric Population Illustrating Risk and Protective Factors

**DOI:** 10.3390/bs14050386

**Published:** 2024-05-03

**Authors:** Mara Stockner, Barbara Plattner, Marco Innamorati, Alex Hofer, Iuliia Burian, Martin Fronthaler, Giancarlo Giupponi, Markus Huber, Christian Macina, Verena Perwanger, Roger Pycha, Gerd Schaller, Andreas Conca

**Affiliations:** 1 Faculty of Medicine and Psychology, Sapienza University of Rome, 00185 Rome, Italy; mara.stockner@uniroma1.it; 2 Department of Psychiatry, Sanitary Agency of South Tyrol, General Hospital of Bolzano, 39100 Bolzano, Italy; barbara.plattner@sabes.it (B.P.); andreas.conca@sabes.it (A.C.); 3 Department of Human Sciences, European University of Rome, 00163 Rome, Italy; 4 Department of Psychiatry, Psychotherapy, Psychosomatics and Medical Psychology, Division of Psychiatry I, Medical University Innsbruck, 6020 Innsbruck, Austria; 5 Department of Psychiatry, General Hospital of Merano, Sanitary Agency of South Tyrol, 39012 Merano, Italy; 6 Therapy Center Bad Bachgart, Sanitary Agency of South Tyrol, 39037 Rodengo, Italy; 7 Department of Psychiatry, General Hospital of Brunico, Sanitary Agency of South Tyrol, 39031 Brunico, Italy; 8 Department of Psychiatry, General Hospital of Bressanone, Sanitary Agency of South Tyrol, 39042 Bressanone, Italy

**Keywords:** COVID-19, pandemic, mental health, suicidal ideation, resilience, spiritual well-being

## Abstract

The COVID-19 pandemic has led to an increase in psychological distress in the general population, but contrasting results have been shown regarding its impact on psychological symptoms in clinical and non-clinical samples. Consequently, the aim of the present study was to compare in a longitudinal design (September–November 2020 and February–April 2021) the mental health outcomes of a clinical and a control sample and to determine the implications of various risk and protective factors in this regard. A total of 234 participants from the general population and 80 psychiatric patients took part in the present online study using the following measurements: the Brief Symptom Checklist (BSCL); Three-Item Loneliness Scale (TILS); Resilience Scale-13 (RS-13); and Functional Assessment of Chronic Illness Therapy—Spiritual Well-Being Scale—Non-Illness (FACIT-Sp Non-Illness). The results show an overall decrease in active suicidal ideation as well as “peace”, a subscale of spiritual well-being, as well as increases in passive suicidal activation in the clinical sample, which did not change in the control sample. Psychological symptoms did not significantly change in either group. Significant group effects show an increase in resilience in the clinical sample. Resilience and peace turned out to be protective factors for negative mental health outcomes. However, loneliness, which interestingly increased only in the control sample, was shown to be an overall potential risk factor. Our results highlight the complex implications of the COVID-19 pandemic on the mental health outcomes of different groups in the population, demonstrating the necessity of further research, specifically regarding the risk of active and passive suicidal activation. Highlighted protective factors are discussed in regards to spirituality (i.e., peace), which is not strictly related to religion but rather personal spirituality related to the meaning of situations of one’s life, as well as in terms of mental health interventions.

## 1. Introduction

An increase in psychological distress with the onset of the COVID-19 pandemic, compared to pre-pandemic levels, has been widely reported in the general population [1,2], although the results are incongruent when studying the stability of mental health impairments in follow-ups, showing both continued worsened mental health [3,4] ) as well as a decrease in or reinstatement of initial mental health problems related to the onset of the COVID-19 pandemic [1,2].

Also, when analyzing the impact of the pandemic on psychiatric patients and comparing it with that on the general population, the literature is characterized by rather conflicting results [5,6] For example, Pico-Perez et al. [6] identified the presence of any psychiatric diagnosis to be a risk factor for worsening mental health during the pandemic. Other studies failed to report significantly worse mental health in clinical samples [2,5,7] while in a single study, Hamza et al. [8] reported the worsening of the mental health of participants with no mental health problems, as well as the stability of or even an improvement in participants whose mental health problems began before the COVID-19 pandemic.

Also, studies investigating the impact of COVID-19 on suicide risk have reported incongruent results [9,10,11,12] For example, Iob et al. [13] found suicidal ideation was present in 18% of those studied during the first wave of the COVID-19 pandemic, and some studies [14] found an increase in suicide risk during the first months of the pandemic. Other longitudinal studies failed to find significant changes in suicidal ideation over time [15,16] and Antonelli-Salgado et al. [17] even found a decrease in suicidal ideation between May and July 2020. Several factors potentially associated with suicide risk during the COVID-19 pandemic have been investigated, such as having contracted SARS-CoV-2 (e.g., Batterham et al. [15] reported a 62% increased risk of suicidal ideation) or feeling alone [17] In fact, loneliness has also been associated with psychological distress [7,18,19] and depression [20] during the COVID-19 pandemic.

Resilience has been widely considered a protective factor for adverse health problems [21], and its role has been extensively investigated during the COVID-19 pandemic [22,23,24,25,26,27] For example, a longitudinal study found stability in resilience levels over the pandemic in the general population [22] Other autors [28] even found an increase in resilience in people with chronic illnesses. Resilience has also been shown to be a protective factor for negative health outcomes during the COVID-19 pandemic [21,29] 

An Important protective factor associated with higher resilience to stress in times of difficulty is spirituality [30,31,32] Moreover, a decrease in spirituality during the pandemic in a German sample has been reported [33] along withimportant differences in its trends according to the levels of personal spirituality before the outbreak of the COVID-19 epidemic. Furthermore, Papadopoulo et al. [34] investigated faith as a protective factor for suicidal ideation, and González-Sanguino et al. [35] investigated this variable as a protective factor for general mental health problems. Both reported the protective effect of faith during the COVID-19 pandemic. Moreover, Tutzer et al. [36] reported on the protective effect of having a sense of meaning in one’s life and peacefulness in terms of psychological distress. Considering these results reported in the literature, the aim of this longitudinal study was to investigate mental health (the severity of psychological symptoms and suicidal ideation) in a clinical sample of psychiatric patients and compare it with that of adults from the general population. Furthermore, we aimed to assess the predictive role of potential risk factors such as loneliness, which has been associated with psychological distress and suicidal ideation during the COVID-19 pandemic [17,18] and protective factors such as resilience and spiritual well-being [21,34].We aimed to investigate these aspects at two measurement points.Ie baseline measurement (T0) was chosen to be carried out between September and November 2020, the time period of the COVID-19 pandemic when residents of South Tyrol were initially affected by only few restrictions, which progressively increased until November, when South Tyrol was declared a “red zone” (see Section 2 for a more detailed description). A post-baseline assessment was carried out between February and April 2021 when the COVID-19 rules in South Tyrol were slowly but steadily relaxed.

We hypothesized significant changes in psychological distress with a significant group effect; i.e., we expected stability of psychological symptoms in the sample recruited from the general population and a decrease in psychological distress in the psychiatric patients [8]. Similarly, we also hypothesized an analogous pattern for suicidal ideation and an increase in resilience in the psychiatric patients. Finally, we hypothesized that loneliness at baseline could predict higher levels of psychological distress and more severe suicidal ideation in the post-test, whereas spiritual well-being and resilience at baseline were expected to be associated with lower levels of psychological distress in the post-baseline assessment.

## 2. Method

### 2.1. Participants

The convenience sample from the general population (controls) consisted of 529 adults residing in the autonomous province of South Tyrol who completed the baseline assessment. Two hundred thirty-four also completed the post-test and were included in the analyses (response rate = 44.2%). The following inclusion criteria were applied: age > 18 years old, residing in South Tyrol, and German or Italian language proficiency. Exclusion criterion was being unable to complete the assessment for various reasons, including the rejection of informed consent at any point.

One hundred and twenty adults with a history of mental health disorders who had been hospitalized in a psychiatric institution in South Tyrol in 2019 completed the baseline assessment, and 80 also completed the post-baseline assessment (response rate = 66.7%). Exclusion criterion was being unable to complete the assessment for various reasons, including the rejection of informed consent at any point. Socio-demographic characteristics of the sample are reported in Table 1 as well as prevalence of psychiatric disorders. The most frequent diagnoses in the clinical sample regarded affective disorders (N = 47; 58.8%), followed by substance disorders (N = 6, 7.5%), psychotic disorders (N = 6, 7.5%), and stress disorders (N = 6, 7.5%).

Participants who completed the study and those who dropped out from the study after the baseline for various reasons did not differ in regards to sex (one-way Fisher exact test *p* = 0.43), ethnic group (one-way Fisher exact test *p* = 0.31), or severity of psychological distress (t_639_ = 1.66; *p* = 0.10), although they differed significantly in terms of age (t_647_ = 4.07; *p* < 0.001). Participants who dropped out after the baseline were on average 4 years younger than other participants (41.41 ± 13.20 vs. 45.56 ± 12.75 years).

### 2.2. Procedure

**Figure 1:**
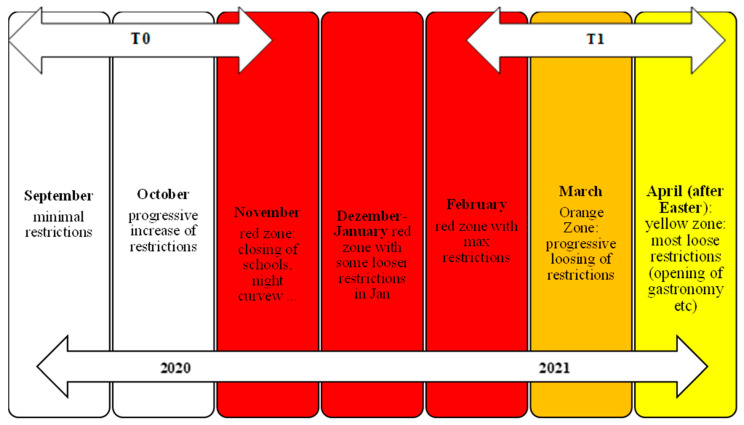
Schematic representation of the two measurement points T0 (baseline) and T1 (post-test) associated with COVID-19 related restrictions.

Recruitment of controls was carried out through the website of the South Tyrolean Health Authority and a snowball system using various social media channels (Whatsapp, email, etc.). The clinical sample was recruited among patients referred to the psychiatric facilities of the South Tyrolean Health Authority (e.g., Psychiatry Department, Mental Health Centres, etc.). All patients who fulfilled the inclusion criteria reported above received a written invitation to participate in the present study. Data were collected with an online questionnaire using the software Computer-based Health Evaluation System (CHES; available at http://www.ches.at (accessed on 19 April 2024) [37]), a web-based program that has been used in various previous studies by the Medical University of Innsbruck. Participation in the study took place through the compilation of an online questionnaire in the period of September–November 2020 (baseline: T0). During that period, Italy entered the second wave of the pandemic with new restrictions after the summer (curfew from 8 p.m. to 5 a.m., closure of sports centers, cinemas, schools, and kindergartens, etc.). Furthermore, on 5 November, South Tyrol entered the red zone, with progressively more restrictions (i.e., closure of restaurants at 6 p.m., closure of shops, etc.). In this regard, it is important to highlight that this period was mainly characterized by a constant change in and reinforcement of emergency measures (see, e.g., Autonome Provinz Bozen [38]) rather than the stable presence of a full lockdown. The post-baseline assessment (post-test T1) took place 11 weeks after baseline, between February and April 2021. During this period of time, South Tyrol was classified as an orange zone: shops were able to reopen, children could attend school in person, one could leave town, etc. (see Figure 1). Thus, the period at T1 was mainly characterized by a constant relaxation of restrictions.

All participants signed informed consent forms online. The research project was approved by the Ethical Committee of the Healthcare Service Alto Adige, and data were collected according to the principles of the Declaration of Helsinki.

### 2.3. Measures

All participants were administered a socio-demographic checklist (sex, age, ethnic group, the presence of any mental health disorders in controls) and a battery of questionnaires: Brief Symptom Checklist [39], Three-Item Loneliness Scale [40], Resilience Scale-13 [41], and Functional Assessment of Chronic Illness Therapy—Spiritual Well-Being Scale—Non-Illness [42]. Patient diagnoses were extracted from medical records.

Brief Symptom Checklist (BSCL). This self-report scale measures 53 symptoms (psychological and physical) across 9 scales: somatization, obsession, social insecurity, depression, anxiety, aggression/hostility, phobic anxiety, paranoid thinking, and psychoticism. Two items (BSCL-9 and BSCL-39) measure active (i.e., “ideas of suicide”) and passive (i.e., “ideas of death”) suicidal ideation. A modified version of the Global Severity Index (GSI) (i.e., average score of all items) was used as a proxy measure of psychological distress. Both the Italian and the German versions demonstrated excellent internal consistency (i.e., Cronbach’s alpha = 0.96; [43]), adequate test–retest reliability, and convergent and divergent validity [39,44]. Cronbach’s alpha in the present sample was 0.97.

Three-Item Loneliness Scale (TILS) is a widely used scale for measuring loneliness derived from the Revised University of California Los Angeles (UCLA) Loneliness scale and consists of the 3 questions: “How often do you miss company?”, “How often do you feel isolated from others?”, and “How often do you feel left out?”. Each item is rated on a Likert-type scale ranging from 0 to 3. The TILS total score ranges from 3 to 9. The psychometric properties of the scale have been shown to be adequate in both the Italian and the German validations [45,46]. More specifically, an adequate Cronbach’s alpha value [47] and adequate convergent and discriminant validation values (i.e., correlations with anxiety, depression, and stress) have been shown [47]. Cronbach’s alpha in the present sample was 0.84.

Resilience Scale (RS-13). Resilience was measured using a short version of the Resilience Scale-25 [48]. Each item is rated on a 7-point Likert scale (from 1 “strongly disagree” to 7 “strongly agree”). The scale has demonstrated satisfactory internal consistency in a German validation study [41]. The 25-item Italian version of the scale has shown a Cronbach’s alpha = 0.84 and adequate test–retest reliability (r = 0.78) as well as adequate concurrent validity by correlations with general health and depression (see [49] ). The 25-item version of the Italian RS has been adapted for the present study to correspond to the German version. Cronbach’s alpha for the present research was 0.93.

Functional Assessment of Chronic Illness Therapy—Spiritual Well-Being Scale (FACIT-Sp Non-Illness). The “non-illness” version of the FACIT scale measures spiritual well-being and consists of 12 items on a 5-point Likert scale [42]. The instructions request that the participants answer each item considering his/her use of spirituality in the context of significant chronic stressors (in our case, the pandemic) instead of illness, as in the original version. The scale consists of 2 subscales: meaning/peace and faith. The FACIT-SP Non-Illness has been translated and linguistically validated in both German and Italian (versions available at www.facit.org). Cronbach’s alphas in the present sample were 0.91 and 0.87, respectively, for peace and faith.

### 2.4. Statistical Analysis

The Statistical Package for the Social Sciences (SPSS) 19.0 was used for all the analyses. General Linear Model for Repeated Measure (RM-GLM) was used to test within-effect of time on outcome measures (i.e., changes in mean scores between the baseline and the post-test) and the interaction effect of the time × group (i.e., changes in outcome measures could be different between controls and patients). Wilks’ lambda (λ) test statistic and its *p*-value are reported as multivariate test of significance. Hierarchical linear regression models were used to evaluate whether resilience, loneliness, and spiritual well-being (i.e., peace and faith dimensions) scores at baseline could predict the criterion (i.e., scores in the post-test for general psychopathology or severity of suicide ideation), while controlling for baseline scores of the dependent variable. First, single-variable regression models were used to evaluate significance of single predictors while controlling for baseline scores of the outcome variable. Second, multivariate regression models were analyzed with variables that were significant in the former step as predictors. All tests were significant for *p* < 0.05.

## 3. Results

### 3.1. Effect of Time and Group on Psychological Distress and Suicidal Ideation

Table 2 shows figures for all of the psychological measures at the baseline and follow-up. When evaluating the effect of time (i.e., changes in variables between the baseline and post-test in the whole sample) and the interaction time × groups (i.e., changes in the controls vs. changes in the patients), we observed that the effect of time was significant for BSCL-9 (Wilks λ = 0.979, F_1;312_ = 6.77, *p* = 0.01; η_p_ = 0.02), BSCL-39 (Wilks λ = 0.968, F_1;312_ = 10.23, *p* = 0.002; η_p_ = 0.03), and peace (Wilks λ = 0.985, F_1;312_ = 4.80, *p* = 0.03; η_p_ = 0.02) items, but not for GSI scores (Wilks λ = 0.995, F_1;304_ = 1.48, *p* = 0.22; η_p_ = 0.005), resilience (Wilks λ = 0.995, F_1;304_ = 1.52, *p* = 0.22; η_p_ = 0.005), loneliness (Wilks λ = 1.00, F_1;312_ = 0.002, *p* = 0.96; η_p_ < 0.001), or faith (Wilks λ = 0.994, F_1;314_ = 1.77, *p* = 0.18; η_p_ = 0.006). The average BSCL-9 scores decreased, and the BSCL-39 and peace scores increased from the baseline to the post-test.

**Figure 2:**
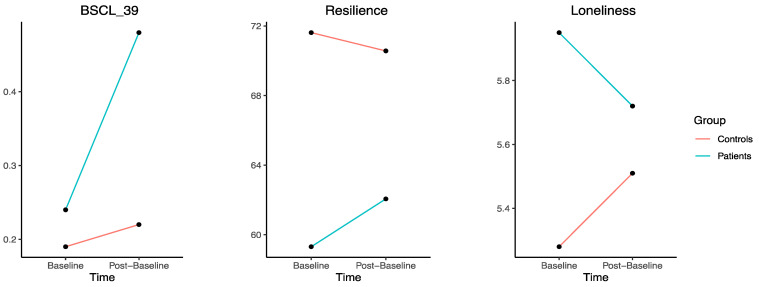
Change in marginal means between baseline and post-baseline for BSCL-39, resilience (RS-13), and loneliness (TILS), for both the control sample and the clinical sample (patients).

When evaluating the possible effect of groups on changes from the baseline to the post-test (group × time effect), we observed that the interaction group × time was significant for BSCL-39 (Wilks λ = 0.982, F_1;312_ = 5.73, *p* = 0.02; η_p_ = 0.02), resilience (Wilks λ = 0.976, F_1;310_ = 7.56, *p* = 0.006; η_p_ = 0.02), and loneliness (Wilks λ = 0.985, F_1;312_ = 4.75, *p* = 0.03; η_p_ = 0.02) (see Figure 2), but not for GSI scores (Wilks λ = 1.00, F_1;304_ = 0.001, *p* = 0.97; η_p_ < 0.001), BSCL-9 (Wilks λ = 0.996, F_1;312_ = 1.39, *p* = 0.24; η_p_ = 0.004), peace (Wilks λ = 0.998, F_1;315_ = 0.61, *p* = 0.43; η_p_ = 0.002), or faith (Wilks λ = 0.995, F_1;314_ = 1.71, *p* = 0.19; η_p_ = 0.005). Thus, levels of loneliness increased significantly from the baseline to the post-test (t_234_ = 2.22, *p* = 0.03), and the scores for BSCL-39 and resilience did not change significantly (*p* > 0.05) in the controls. Conversely, levels of loneliness did not change significantly (*p* = 0.22), and both resilience (t_234_ = 2.51, *p* = 0.01) and BSCL-39 scores (t_234_ = 2.28, *p* = 0.03) increased significantly in the patients.

### 3.2. Predictors of Psychological Distress and Suicidal Ideation in the Post-Test

Our single hierarchical models, in which the GSI scores in the post-test were considered as a criterion and GSI scores at the baseline were controlled for, indicated that the inclusion of loneliness (R^2^ change < 0.001, *p* = 0.89), peace (R^2^ change = 0.003, *p* = 0.08), or faith (R^2^ change < 0.001, *p* = 0.60) was not able to increase the explained variance of the criterion. Only the inclusion of resilience (R^2^ change = 0.01, *p* = 0.001) was able to increase the explained variance of the criterion, and resilience was significantly and independently associated with the GSI scores in the post-test (beta = −0.12, t = 3.39, *p* = 0.001). More specifically, this indicates that higher resilience levels lead to a decrease in GSI scores.

### 3.3. Predictors of Active Suicidal Ideation in the Post-Test

Our single hierarchical models, in which the BSCL-9 scores in the post-test were considered as a criterion and the BSCL-9 scores at the baseline were controlled for, indicated that the inclusion of loneliness (R^2^ change = 0.002, *p* = 0.36) and faith (R^2^ change = 0.007, *p* = 0.051) was not able to increase the explained variance of the criterion. Only the inclusion of resilience (R^2^ change = 0.03, *p* < 0.001) and peace (R^2^ change = 0.03, *p* < 0.001) as predictors was able to increase the explained variance of the criterion. When including all the significant variables in a multivariate hierarchical model, they were able to explain only an additional 3% of the variance in the criterion (*p* < 0.001), and only peace was a significant and independent predictor of BSCL-9 scores in the post-test (beta = −0.15, t = 2.08, *p* = 0.04). This latter result indicates that higher scores of peace were associated with decreased BSCL-9 scores in the post-test.

### 3.4. Predictors of Passive Suicidal Ideation

Our single hierarchical models, in which the BSCL-39 scores in the post-test were considered as a criterion and Ithe BSCL-39 scores at the baseline were controlled for, indicated that the inclusion of any of the following variables was able to increase the explained variance of the criterion: resilience (R^2^ change = 0.05, *p* < 0.001), loneliness (R^2^ change = 0.04, *p* < 0.001), peace (R^2^ change = 0.07, *p* < 0.001), and faith (R^2^ change = 0.02, *p* = 0.009). When including all the significant variables in a multivariate hierarchical model, they were able to explain only an additional 8% of the variance of the criterion (*p* < 0.001), and only peace was a significant and independent predictor of BSCL-39 scores in the post-test (beta = −0.19, t = 2.40, *p* = 0.02), indicating higher levels of peace are associated with decreased BSCL-39 scores in the post-test.

## 4. Discussion

Investigating risk and protective factors, the present study aimed at evaluating the changes in psychological distress and suicidal ideation in the general population and a clinical sample using a longitudinal design. Our results regarding psychological symptoms are in line with those of the literature, confirming the expected stability of psychological distress across our observations. However, we did not observe the expected decrease in the clinical sample (RIF). While one could argue this is due to the observed low GSI scores in our sample, our scores seem in line with those reported in other (clinical) studies [50,51] and studies that likewise did not find significant changes in clinical samples [5,7]. These studies argue that this finding could be due to psychiatric patients generally being less affected by the specific consequences of the COVID-19 pandemic (see also [52]). Our results regarding active and passive suicidal ideation only partially confirmed our initial hypotheses. While we found active suicidal ideation to decrease from the baseline to the post-test, we found passive suicidal ideation to increase significantly (in the clinical sample). While there is a lack of literature on the differential impact of the COVID-19 pandemic on active vs. passive suicidal ideation, it is possible to explain our results in light of their conceptual difference (as discussed, e.g., in [53]). ). Active suicidal ideation is, unlike passive ideation, characterized by a sense of hopelessness that derives from the sense that a situation is not changing. This is clearly attributable to our baseline measurement which was carried out just at the beginning of the second “lockdown” of the COVID-19 pandemic (i.e., when there was a progressive increase in restrictions, such as closed restaurants, a night curfew from 8 p.m. to 5 a.m., etc.). This period was potentially characterized by uncertainty and negative or hopeless feelings in regards to the beginning of the second COVID-19 wave, as the Italian population had already experienced a very strict lockdown during the first wave, which involved the restriction of movement except for emergencies, essential work, and health appointments for over two months. Thus, the start of the second COVID-19 wave, when people did not know if the same strict lockdown would be applied, might have been characterized by a higher level of uncertainty compared to that of the post-test period, which was characterized by progressively fewer restrictions (i.e., shops and schools were open, people were able to leave town, etc.) and fewer feelings of hopelessness derived an unchangeable situation.

On the other hand, passive suicidal ideation, which is generally also associated with mental health problems or associated risk variables [54], increased according to our observations, possibly being an indicator of an increase in general distress due to the prolonged stressful situation. Even though the situation was no longer an acute emergency in the “red zone”, the pandemic and (lighter) restrictions persisted. This is further in line with other findings. Sapara et al. [55] also found that passive suicidal ideation increased in response to the pandemic ( during the first lockdown) and illustrated that individuals with existing mental health problems (e.g., higher anxiety levels) are at a higher risk for this to develop. This supports our findings of a significantly higher increase in passive suicidal ideation in the clinical group (see also [56]). Due to the important clinical and behavioral correlates of passive suicidal ideation [54] and the lack of studies in this regard, there is need for further studies that more specifically investigate Ithe impact of prolonged stress, such as a pandemic, on these two related (but distinct) dimensions.

Regarding our hypothesized pre–post changes, our results further confirmed that resilience increased between the baseline and the post-test in the clinical sample. This is in line with the literature, which has illustrated the increase in resilience in vulnerable groups [28] and the stability of resilience in response to the pandemic in the general population [22] Whereas this result might seem in contradiction with the increase in passive suicidal ideation in the clinical sample, these findings are most likely due to the shift from active to passive suicidal ideation according to our observations, which is accompanied by an increase in resilience. Furthermore, our results confirm that resilience is a protective factor against the severity of psychological symptoms as well as a predictor of suicidal ideation. Also, this has been similarly found by other authors who have shown that low levels of resilience were associated with more psychological symptoms during the COVID-19 pandemic [57,58] and that resilience was a protective factor against various negative mental health outcomes [21,29]

Our results regarding spirituality, which was expected to be a protective factor, show first of all an increase between the baseline and post-test (more specifically, an increase in the subscale “peace”, which relates to having a sense of meaning and peace in one’s life). The literature is lacking in this regard, and the few studies that have been conducted thus far did not find any significant changes in spiritual-well being [35,59]. One possible explanation for our result could be the progressive adaptation of the participants to the pandemic, combined with the relaxation of pandemic-related restrictions during the post-test (our post-test was carried out much later (February–April 2021) than the above-cited studies (spring/summer 2020)). On the other hand, the fact that both of the subscales “peace” and “faith” were able to predict psychological symptoms and that peace was an independent protective factor for suicidal ideation is in line with the results in the literature. For example, González-Sanguino et al. [35] showed spiritual well-being to be one of the main predictors and protective factors of various psychopathologies, playing a significant role as an emotional regulation mechanism. On the other hand, contrasting studies (discussed above) have illustrated that spirituality or faith was a risk factor for poor mental health outcomes during the pandemic [60,61], as it was impossible to carry out religious practices in, e.g., churches during the pandemic. Our findings on spirituality, however, are not strictly related to religion, leading us to hypothesize that personal spirituality, which is trying to find meaning in one’s situation in life, rather than religious spirituality should be considered a protective factor. 

Finally, our analysis on loneliness, an expected risk factor for psychological distress and suicidal ideation, showed an increase over time but only in the control sample. Hamza et al. [8] claim this could be due to psychiatric patients generally being more used to loneliness due to their disorders or health conditions, whereas the general population might have suffered more from the COVID-19 restrictions as time went on (see also [62]). We also found loneliness to be a predictor of passive suicidal ideation; this has been confirmed by other authors (see [17] in terms of depression, including suicidality and see [20,35] On the other hand, the loneliness scores at the baseline do not seem to have been a risk factor for psychological distress, possibly due to the stable levels of these scores.

Our study is characterized by various methodological limitations. First of all, the small sample size and differences between the two groups have to be highlighted, leading to a high level of heterogeneity regarding clinical diagnoses, especially in the clinical group (see Table 1). As the latter are characterized by different symptoms, they might be influenced differently by the pandemic, but due to the small sample size for each category, distinct analyses were not possible. Furthermore, the chosen testing periods might limit the present results, as laws and restrictions due to COVID-19 changed quickly and the participants could fill out the questionnaires over a period of two months, respectively, with a time interval of 11 weeks between the baseline and post-test. In this regard, potential seasonal effects on affective disorders or psychopathological symptoms [63] were not considered. A further limitation is given by the impossibility of comparing the data with pre-pandemic data in order to understand the specific impact of the COVID-19 pandemic on the samples. Finally, one main concept investigated in the present study, passive vs. active suicidal ideation, was assessed with one item, respectively, which could limit the reliability of the results. Thus, future studies are needed to investigate the (potentially) different trajectories of passive and active suicidal ideation, two related but distinct constructs [64], in response to stressful periods, such as a pandemic, both in healthy and clinical samples and with more robust methodologies. Finally, our findings on the highlighted risk factors as well as protective factors might contribute to mental health interventions (both primary and secondary) in situations like the COVID-19 pandemic, especially regarding the concept of “peace”, which has been widely investigated in patients with organic illnesses [65]. however, to our knowledge, few studies have focused on psychiatric patients or its importance during stressful periods, such as pandemics.

## 5. Conclusions

The present study illustrated using a longitudinal design the impact of risk and protective factors on the mental health of the general population as well as psychiatric patients during the COVID-19 pandemic. Our findings highlight the psychological stability and decrease in active suicidal ideation in the general population. The latter change was also observable in the clinical sample, together with an increase in passive suicidal ideation. This “switch” from active to passive was accompanied by an analogous increase in resilience. The most significant protective factors for psychological distress and suicidal ideation were “peace”, as part of spiritual well-being, together with resilience.

## Figures and Tables

**Table 1:** Socio-demographic characteristics of the sample.

Variables		Controls N = 234	Patients N = 80	Test	*p*
		N (%)	N (%)		
Sex					<0.001
Males		54 (23.1)	36 (45)		
Females		180 (76.9)	44 (55)		
Age—M (SD)		45.62 (12.24)	45.39 (14.23)	t (312) = −0.140	0.88
Ethnic group					
German		214 (91.5)	58 (72.5)		<0.001
Italian		17 (7.3)	22 (27.5)		
Ladin		1 (4)	0 (0)		
Other		2 (9)	0 (0)	χ^2^ (3) = 23.15	
Mental disorder	yes	27 (11.5)	- (-)		-
Diagnosis					
Substance disorders		- (-)	6 (7.5)		
Psychotic disorders		- (-)	6 (7.5)		
Affective disorders		- (-)	47 (58.8)		
Anxiety disorders		- (-)	3 (3.8)		
Obsessive-compulsive disorder		- (-)	2 (2.5)		
Stress disorders		- (-)	6 (7.5)		
Somatic disorders		- (-)	1 (1.3)		
Neurotic disorders		- (-)	1 (1.3)		
Sleep disorders		- (-)	4 (5)		
Personality disorders		- (-)	1 (1.3)		
Impulse control disorders		- (-)	2 (2.5)		
Behavioral disorders (childhood)		- (-)	1 (1.3)		

**Table 2:** Descriptive results.

Variable		T0: M (SD)	T1: M (SD)
GSI	Control	0.57 (0.53)	0.54 (0.55)
	Patients	0.85 (0.70)	0.81 (0.67)
BSCL-9	Controls	0.18 (0.55)	0.13 (0.43)
	Patients	0.51 (0.87)	0.39 (0.72)
BSCL-39	Control	0.19 (0.49)	0.22 (0.59)
	Patients	0.24 (0.60)	0.48 (0.94)
RES-13	Control	71.62 (11.48)	70.57 (14.17)
	Patients	59.31 (15.11)	62.06 (13.47)
TILS	Control	5.28 (1.88)	5.51 (1.86)
	Patients	5.95 (1.95)	5.72 (1.94)
FACIT Peace	Control	2.98 (0.76)	3.03 (0.73)
	Patients	2.32 (0.93)	2.42 (0.89)
FACIT Faith	Control	1.65 (1.06)	1.76 (1.08)
	Patients	1.48 (1.15)	1.48 (1.08)

Note. GSI = Global Severity Index of the Brief Symptom Checklist; BSCL = Brief Symptom Checklist; BSCL-9 = active suicidal ideation; BSCL-39 = passive suicidal ideation; RES-13 = Resilience Scale-13; TILS = Three-Item Loneliness; FACIT = Functional Assessment of Chronic Illness Therapy—Spiritual Well-Being Scale—Non-Illness.

## Data Availability

The dataset is available upon request from the authors.
